# Purification, identification, and *in silico* screening of a multifunctional octapeptide from *semen armeniacae* glutelin-2 hydrolysates: restraining mechanisms to Keap1 and ACE, stability, and ferrous-transport efficiency

**DOI:** 10.3389/fnut.2025.1571161

**Published:** 2025-04-04

**Authors:** Ziqing Jin, Ling Dang, Yan Li, Chen Feng, Xinling Song, Zhihui Wei, Jie Liu, Hao Wang, Yichan Zhang

**Affiliations:** ^1^Food Science College of Shanxi Normal University, Taiyuan, China; ^2^Shanxi Technology and Business University, Taiyuan, China

**Keywords:** *semen armeniacae* glutelin-2 octapeptide, dual enzymolysis, angiotensin-I-converting enzyme, Keap1, inhibition mechanisms, antioxidant, ferrous absorptivity

## Abstract

**Introduction:**

*Semen armeniacae* is a traditional homologous material of medicine and food, but data on its multifunctional peptides are little.

**Methods:**

In this study, *semen armeniacae* glutelin-2 was hydrolyzed by alcalase and trypsin assisted with ultrasound. Antihypertensive and antioxidant peptides with ferrous-binding activity were isolated, identified, and *in silico* screened from the hydrolysates, and the action mechanisms against Keap1 and angiotensin-I-converting enzyme (ACE), gastrointestinal stability, and ferrous-binding capacity were studied.

**Results and discussion:**

After Sephadex G-15 isolation, electrospray ionization mass spectrometry, and AHTpin and Peptide Ranker database screening, a safe multifunctional octapeptide: Pro-Val-Asp-Phe-Ala-Gly-Phe-Tyr (PVDFAGFY), was obtained. The capacities of PVDFAGFY to restrain ACE, chelate ferrous ions, and quench hydroxyl radical were IC_50_:105.61 μmol/L, 11.67 mg/g, and 97.67%, respectively. PVDFAGFY restrained ACE via competitively linking to its catalytic (His383) and/or crucial binding sites (Gln281, Lys511, Tyr523, Tyr520, or Ala354), and it can inhibit the Keap1-Nrf2 interaction by binding to 6 residues of Keap1. Ferrous ions were primarily chelated by *γ*-hydroxyl, carboxyl, and/or amino groups of PVDFAGFY via ionic forces. Gastrointestinal hydrolysis did not decrease the capacity of PVDFAGFY to antioxidant and restrain ACE (*p* > 0.05). The ACE inhibition model and activity of PVDFAGFY were not altered by iron chelation; however, PVDFAGFY-ferrous chelate showed lower hydroxyl and ABTS radical quenching capacity and ferric reducing ability than PVDFAGFY (*p* < 0.05). The gastrointestinal stability and transmembrane absorption of ferrous ions were increased by PVDFAGFY (*p* < 0.05). Thus, PVDFAGFY may be exploited as ingredients of hypotensive, antioxidant, and/or iron supplementary agents, but *in vivo* antioxidant and hypotensive efficiencies need further study.

## 1 Introduction

Oxidative stress brings damage to cell membrane biomacromolecules, causing apoptosis, organic damage, and diseases in sequence (1). During food storage, oxidative browning and damage are main inducements for food deterioration in nutrition, sensory quality, and safety (2). The Keap1-Nrf2-ARE system is an important defense mechanism to mitigate oxidative stress and maintain body health (3, 4). In this system, Keap1 negatively regulates nuclear factor erythroid 2-related factor 2 (Nrf2) which controls the expression of downstream antioxidant enzymes and cytoprotective genes (5). Peptides that can cause the separation of Keap1 and Nrf2 can promote the expression of cytoprotective genes and antioxidant enzymes and thus lower cellular oxidative pressure (6). Recently, due to safety, economy, and availability, food-derived antioxidant peptides and their efficiency in food preservation and the prevention of cardiovascular diseases have received much attention (7, 8). Apart from oxidative stress, hypertension is the primary inducement for cardiovascular diseases (9). Approximately one-fourth of the people suffer from hypertension and hypertension complications such as stroke, cerebral hemorrhage, and coronary heart disease in the world (10). Although the specific mechanisms of hypertension remain unclear, the crucial role of angiotensin-I-converting enzyme (ACE) in controlling blood pressure has been clinically confirmed (11, 51). It has been confirmed that peptides will offer *in vivo* hypotensive efficiency if they have capacity to restrain Keap1and ACE, gastrointestinal stability, and transmembrane absorption (2, 3, 12). Furthermore, there is a zinc tetrahedron in catalytic center of ACE. Several chemical groups in peptides, especially the phenolic hydroxyl, carboxyl, and amino groups, can influence the catalytic center of ACE by ionic forces and offer potential hypotensive efficiency (51). Recently, ACE-inhibitory peptides identified from various plant, animal, and microbial food receive more attentions for their potential antihypertension, economy, and few side effects (13–15). Another feature of these peptides is that they all have good metal chelation ability (16). Approximately 240 million people worldwide are threatened by iron deficiency anemia (17). The predominant reasons for iron deficiency are the poor stability of iron in the gastrointestinal tract, low absorption rate, and the strong iron demand during pregnancy and infancy. Acid–base changes, oxidative reaction, oxide, and nutrients in the digestive fluid of food, such as phytic acid, fiber, and metal ions, all can convert food ferrous to trivalent iron, which cannot be absorbed by intestinal cells (18). Compared with synthesized antioxidants, hypotensive drugs, and customary ferrous supplements (ferrous chloride and lactate), food-derived antioxidant and antihypertensive peptides, and peptide-iron chelates are better at safety, stability, scalability, and absorptivity (19–21); however, their structure–activity relationship, specific action mechanisms, and functionalities *in vivo* have been scarcely conducted (22). Furthermore, increasing studies have studied peptides with single functionality, such as antioxidant, antihypertensive, or iron-supplementary peptides, from different food resources (5, 23), but food-derived multifunctional peptides, especially those have significant efficacy to mitigate oxidative stress, decrease blood stress, and fortify iron, are rarely studied (17, 23). With respect to releasing bioactive peptides from food proteins, the combination of exo-protease, endoprotease, and ultrasound has been proven to be an effective way (24, 25), but this method is rarely used in preparation of multifunctional peptides.

Bitter almond (*Semen Armeniacae Amarum*) is widely used in almond oil, amygdalin, food excipients, and traditional Chinese medicine (1). In China, it is used to treat cough and asthma, chest full of phlegm, and intestinal dryness constipation (26). Its oil processing byproduct has a protein content of approximately 45 g/100 g, and glutelin-2, albumin, and globulin account for 22.15, 45.76, and 41 g/100 g almond protein, respectively (27). The functionalities of *semen armeniacae* peptides, such as hypoglycemic, antibacterial, antihypertensive, and anti-ultraviolet radiation activities, have been studied (1, 28, 29). However, there are little data referring bioactivities of bitter almond glutelin-2. Defatted bitter almond glutelin-2 hydrolysates (BAG-2H) have been proven to be a resource of multifunctional peptides because of its ACE restraining capacity (54.22% ± 3.00%), hydroxyl and ATBS radical quenching activity (43.07 and 60.05%, respectively), and ferrous-binding ability (4.79 ± 0.09 mg/g). In this study, antihypertensive and antioxidant peptides with ferrous-binding activity were isolated, identified, and *in silico* screened from BAG-2H in this study. Another purpose was to investigate the action mechanisms against Keap1 and ACE, gastrointestinal stability, and ferrous chelating coordination and transmembrane absorption. New ideas and strategy for the development of foodborne multifunctional peptides will be provided by this study.

## 2 Materials and methods

### 2.1 Materials

Apricots Garden Oil Processing Co. (Guangling, China) provided the bitter almond (*Semen Armeniacae Amarum*) expeller powder that was produced on 12 July 2023. Pepsin (1:100,000 U*·*g^−1^) and trypsin (1:3000 U·g^−1^) were purchased from Nanjiang Enzymatic Reagent Co., Ltd. (Nanning, China). Kunming Zoology Research Institute (Kunming, China) provided Caco-2 cells, fetal bovine serum, and Hank’s Balanced Salt Solution. Captopril, Dulbecco’s modified Eagle’s medium, alcalase (from *Trichoderma Viride G*, 1.0 × 10^5^ U/g), and glutathione were obtained from Peisu Biotech. Co. Ltd. (Shanghai, China). Rabbit lungs ACE (0.1 U) was obtained from Sigma-Aldrich (St. Louis, MO, USA). All analytical grade reagents, such as acetonitrile, potassium persulfate, and potassium ferricyanide, were obtained from Keoumi Chemicals Factory (Shanghai, China).

### 2.2 Extraction of bitter almond glutelin-2

Bitter almond expeller was heated by blast air at 45 ± 1°C for 7 h using a 78HET-B blast drier (Shaoxing Drier Factory, Shaoxing, China) and then smashed and sifted through a 60-mesh screen (DY-200, Dayong Vibration Equipment Co. LTD, Xinxiang, China) (30). Next, 300 mL of petroleum ether II were used to degrease the bitter almond expeller powder (60 g) at 205 rpm and 35 ± 1°C using an EDZF-C002 thermostatic vibrator (Nantong Vibration Shaker Co., Nantong, China) for 150 min. After filtration with a 113-25-Whatman paper, the residue on the filter paper was collected to obtain the defatted bitter almond expeller. Then, the deoiled bitter almond was thoroughly dispersed in 0.1 mol/L of NaOH (1:25, m/v). After 120 min of stirring at 175 r/min and 35°C using the EDZF-C002 thermostatic vibrator, filtration of the dispersions was conducted on a 113-25-Whatman paper. The percolate was used for centrifugation (13,700 × g, 4°C, and 15 min) on a centrifugal machine (H1750R, Xiangyi Centrifuge Development Co., Ltd., Changsha, China). The supernatant solution was poured into a dialysis bag with cutoff weight of 7,500 Da (Sanjiang Filtration Material Co. Ltd., Chengdu, China), sealed, and dialyzed against deionized water (dH_2_O) at 4°C (31). The dH_2_O was changed every 4 h. After 48 h, the dialysate was centrifuged (13,700 *× g*, 15 min), and the pellet was lyophilized employing an EHI-220D lyophilizer (Lingling Lyophilize Instrument Co. Ltd., Wuchang, China) to obtain bitter almond glutelin-2.

### 2.3 Preparation and purification of BAG-2H peptides

Ultrasonic treatment of bitter almond glutelin-2 solution (20 mg/mL, pH7.8) was conducted using an ultrasonic cell crusher (UH3000-AIO, Shanghai Ouhe Ultrasonic Co., Ltd., Shanghai, China). The ultrasonic power, temperature, frequency, and treated time were 400 W, 50°C, 59 kHz, and 25 min, respectively (25). The bitter almond glutelin-2 solution was taken away the reaction kettle and cooled to 25 ± 1°C, and 40 mg alcalase was added and then shaken at 50°C, 220 rpm, and pH 8.5 in a ZDF-C04 thermostatic vibrator for 90 min (30). Next, the proteolysis dispersion was adjusted to pH 7.0 ± 0.1, and trypsin (0.02 g) was added and shaken at 37°C with a shaking rate of 220 rpm for 1 h. After enzyme deactivation (100°C, 10 min), the dispersion was cooled to 25 ± 2°C and then centrifuged (13,700 × *g*, 10 min). The super solution was pooled and freeze-dried with the EHI-220D lyophilizer, and then, bitter almond globulin hydrolysates (BAG-2H) were obtained. BAG-2H’s degree of hydrolysis determination was conducted citing the formaldehyde titration method (32), whereas the protein content was measured following the Kjeldahl method (33).

Next, the purification of BAG-2H is a two-step process: first, BAG-2H solution (1 g/L) was subjected to ultra-filtration on an ultra-membrane (filter aperture of 220 nm, Jingfei Membrane Equipment Factory, Luzhou, China). Next, the ultra-filtrate solution (2 mL) was chromatographically purified citing the modified procedures from Li et al. (30). Pre-balanced G-15 gel was loaded into a HS-MG-125 chromatographic column (Huasheng Chromatography Tech. Co., Ltd., Wuxi, China). After the BAG-2H ultra-filtrate solution has completely sunk into the column plane, the gel chromatographic column was sealed and eluted using dH_2_O with a constant velocity of 2.4 mL/min and monitor wavelength of 220 nm. An automatic collector (BT-160I, Thirdli Collection Equipment Co., Ltd., Guangzhou, China) was used to collect the elution, and the collection tube was changed every 5 min (34). After 400 min, the profiles of absorbance at 220 nm against elution time were drawn, and the separation fractions were freeze-dried using the EHI-220D lyophilizer. The ACE-inhibitory, ferrous-binding, and hydroxyl radical quenching abilities of the separation fractions were detected citing the hippuric acid (50), *β*-deoxyribose oxidation (35), and *o*-phenanthroline methods (31), respectively. During screening of the separation fractions, ACE-inhibitory and hydroxyl radical quenching abilities were the primary indexes, and the amino acid sequences of the selected fraction were analyzed.

### 2.4 Restraining capacity and kinetics against ACE

The hippuric acid method (50) was used to measure the samples’ restraining capacity against ACE citing the same procedures from Zheng et al. (34). The absorbance of the reaction solution at 228 nm was the hippuric acid content and was proportional to ACE activity; therefore, the restraining capacity of samples toward ACE was defined as the percentage reduction in the absorption at 228 nm between the control and sample groups. In addition, the ACE-inhibitory kinetic by BAG-2H peptides (0.011–0.055 mmol/L) was investigated employing Lineweaver–Burk plot (36). The concentration of substrate (*N*-hippuryl-L-histidyl-L-leucine) was 0.13–1.32 mmol/L. ACE-inhibitory mode was determined from the influence of inhibitors on the Michaelis constant (*K_m_*) and maximum velocity (*V_m_*) of the Michaelis–Menten kinetic curves.

### 2.5 Antioxidant ability

#### 2.5.1 Ferric reducing ability

Ferric reducing ability was defined as the ability of antioxidants to provide protons, which was administrated according to the Prussian blue method (2). A total 1 mL of BAG-2H peptide solution (100 μg/mL, dissolved in 0.2 mol/L of phosphate buffer, pH 6.8) was reacted with potassium ferricyanide (10 mg/mL, 4 mL) in the EDZF-C002 thermostatic vibrator (50°C, 220 rpm) for 30 min. The reaction solution was cooled to 25 ± 1°C and precipitated using an equal volume of 100 mg/mL trichloroacetic acid for 10 min. Then, the reaction solution was centrifuged at 3000 *× g* for 25 min, and 1 mL of the upper solution was pipetted and reacted with 1 mg/mL of FeCl_3_ (1.2 mL) to produce Prussian blue (potassium ferrocyanide). The concentration of potassium ferrocyanide produced was positively correlated with the ferric reducing ability of the sample (37), which was determined at 700 nm using a HD-UV90 ultraviolet–visible spectrophotometer (Holder Technology Co. LTD, Weifang, China).

#### 2.5.2 Free radical quenching ability

The free radical quenching ability of samples, including superoxide, 2,2′-hydrazine-bis (3-ethylbenzothiazolin-6-sulfonic acid) diamine salt cation (ABTS^+^), and hydroxyl radicals, was determined using the pyrogallol auto-oxidation, potassium persulfate, and *β*-deoxyribose oxidation methods, according to the same procedures from Tyagi et al. (6), Lin et al. (2), and Wang et al. (35), respectively. Comparison was conducted when the samples were replaced by glutathione (0.1 mg/mL).

### 2.6 Ferrous ion-binding capacity

According to the procedures from Xu et al. (31), BAG-2H peptides (1 mg) were dissolved in ultrapure water (32 mL) in a glass conical flask. In total, 3 mL of sodium acetate (0.1 mg/mL) and 1 mL of hydroxylamine hydrochloride (0.1 mg/mL) were added and mixed at 1,200 rpm using a JL-D vortex oscillation (Jinlan Instrument Manufacturing Co., Ltd., Dalian, China) for 6 s. Then, *o*-phenanthroline (2.4 mg/mL, 0.5 mL) was added, and the reaction was started. After 45 min of shaking at 25 ± 1°C and 115 rpm with the EDZF-C002 thermostatic vibrator, the absorbance at 510 nm (*A*) of the reaction solution was measured. By plugging the absorbance value into the equation of *A* = 0.3719*C* + 0.0002 (38), the ferrous ion concentration (*C*) can be calculated. The ferrous ion-binding capacity (mg/g) was quantified from the decrease in ferrous concentration of the reaction solution per BAG-2H peptides’ concentration.

### 2.7 Detection and verification of amino acid sequence

Amino acid sequence was analyzed citing the same procedures from Xie et al. (50) and using an LMS-6100B Hybrid-Triple-Quadrupole liquid–mass tandem mass spectrometry system (Agilent Technologies Inc., California, USA). The mode of electrospray ionization needle was coupled G-1958 in terms of positive, and the analysis was conducted at spray voltage of 4.4 kV, spray flow rate of 50 μL/min, data scanning range of 100–3,000 m/z, AGC target of 5 e^5^, capillary temperature of 360°C, and mass resolution full width at half maximum of 70,000, respectively (50). A PEAKS^®^ Studio 12.5 DeepNovo Peptidome software (Bioinformatics Solutions Inc., Ontario, Canada) was used to process the obtained mass spectrometry data. Peptide identification was accepted if it could be established with a probability >80%. The National Center for Biotechnology Information^1^ was used for verification of the peptide sequences obtained.

### 2.8 *In silico* screening and synthesis

Physicochemical properties and functionalities of BAG-2H peptides, including antihypertension and antioxidant activity, were *in silico* analyzed with the AHTpin^2^ and Peptide Ranker server^3^ databases (39), respectively. The thresholds for antioxidant and hypotensive peptides were the probability value for Peptide Ranker>0.5 and the vector machine software score (VMSS, for AHTpin) > 0, respectively (6, 50). The sequences with potential hypotensive and antioxidant capacity were chemically synthesized according to the standard solid phase way in Dongdan Bioactive Peptides Co. Ltd. (Lianyungang, China). The purity of the synthesized peptides>99.5% and its abilities to bind to ferrous ions, restrain ACE, and quench hydroxyl radical were investigated following the methods of Xu et al. (31), Xie et al. (50), and Wang et al. (35), respectively.

### 2.9 Allergenicity and toxicity analysis

Sensitization and toxicity of BAG-2H peptides were *in silico* analyzed with AlgPred^4^ and ToxinPred^5^ databases, respectively (40). The threshold value for AlgPred prediction was 0.4 (34). The predicted values by ToxinPred “–0.5,” “0,” and “+0.5” meant non-toxic, no matched, and toxic peptides, respectively (40).

### 2.10 Molecular docking

A TORSEP SLYBYII 2.0 software (SRULEXF-SCORK, Troesp Co., Missouri, USA) with Kuntz-D score, scoring function, total score (T-score), and consistency score (C-score) was used to determine the potential specific interaction modes of BAG-2H peptides with key antioxidant and antihypertensive targets in Keap1 (ID:2FLU, from https://www.rcsb.org/) and ACE (PDB-108A, from rcsb.org/structure), respectively. Molecular docking conformations were acceptable when their C-score and T-score were more than 4.0 and 6.0, respectively (22).

### 2.11 Ferrous-chelating mode of BAG-2H peptides

The synthesized BAG-2H peptides with hypotensive and antioxidant activities were dissolved in ultra-pure water (2.75 mg/mL, 5 mL) and then mixed with double volume of ferrous chloride solution (0.26 mol/L) and 175 μmol/L of Vitamin C (0.45 mL). The reaction solution was adjusted to pH 5.4 and shaken at 35 ± 1°C in the EDZF-C002 thermostatic vibrator with stirring rate of 225 rpm for 47 min (31). After 20 min of centrifugation (7,300 × *g*), the pellet was discarded and supernatant liquid was precipitated by four times the volume of anhydrous ethanol. BAG-2H peptides ferrous chelates were got after the sediment was freeze-dried employing an EHI-220D lyophilizer (Lingling Lyophilize Instrument Co. Ltd., Wuchang, China). Afterward, dry KBr (30 mg) was thoroughly mixed with the BAG-2H peptides (1.5 mg) or their ferrous chelates (1.5 mg) under NL-3C Infrared baking lamp, respectively, and then pressed into 1–2 mm sheets (21). Those sheets were analyzed with an ILDA-20 Fourier-transform infrared (FT-IR) spectrometer (Hengchuanglida Precision Instrument Co. Ltd., Tianjin, China) at wavenumber range of 4,000–400 cm^−1^.

### 2.12 Gastrointestinal stability of BAG-2H peptides and their ferrous chelates

As described by Wu et al. (41), the simulative gastric digestive fluid consisted of sodium chloride (0.18 mol/L), ultrapure water (180 mL), and 0.40 mg/mL of pepsin. The simulation intestinal digestive juice was composed of NaHCO_3_ (0.625 g/mL), bile salt (3 g/100 mL), pancreatin (0.35 mg/mL), and 180 mL ultrapure water. In a glass triangle flask, 300 mg of the antihypertensive and antioxidant BAG-2H peptides, 60 mL of ultrapure water, and 150 mL of the simulative gastric fluid were mixed thoroughly. The flask was placed at 37°C in an EDZF-C002 thermostatic vibrator with shaking rate of 140 rpm. After 90 min, 180 mL of the simulative intestinal hydrolysis fluid was added and stirred at 37°C for 2 h. To stop the hydrolysis, the digestion solution in the flask was boiled for 6 min and then cooled by running water to room temperature. The ACE inhibition capacity and antioxidant ability of the treated BAG-2H peptides were detected and compared to those of the undigested BAG-2H peptides.

The stability of the ferrous chelate of BAG-2H peptides under gastrointestinal digestion was studied as follows: 2.75 mg BAG-2H peptide-ferrous chelate was subjected to simulative gastric fluid hydrolysis at pH 1.9 ± 0.1, 175 rpm, and 37°C using the EDZF-C002 thermostatic vibrator for 90 min (41). Next, the intestinal digestive fluid was added, and the digestion was continued at pH 6.8 for 2 h in the EDZF-C002 thermostatic vibrator (90 min, 175 rpm). During the gastrointestinal digestion, ferrous gastrointestinal stability was represented by soluble ferrous content in the digestive dispersion, which was determined every 30 min following the *o*-phenanthroline method (38), and the sample data were compared with those of ferrous lactate and ferrous chloride (0.1 mg/mL).

### 2.13 Ability to promote iron transmembrane absorption

In a 24-well transwell culture plate, Caco-2 cells (0.75 × 10^6^ cells/cm^2^) were cultivated by Dulbecco’s modified Eagle’s medium that contained fetal bovine serum (20 g/L), streptomycin (1 μg/μL), penicillin (1 μg/μL), and neomycin (1 μg/μL) and refreshed every 48 h. After 12–14 d of incubation at 5% CO_2_ and 37°C, the transepithelial electrical resistance was more than 400 *Ω*·cm^2^ and a monolayer Caco-2 cell mode was formed. Afterward, the medium was removed, and Hank’s Balanced Salt Solution was added. After 30 min of cultivation at 5% CO_2_ and 37°C, Hank’s Balanced Salt Solution was removed from the apical side and BAG-2H peptide-ferrous chelates (320 μg/mL) were added and then cultivated at 5% CO_2_ and 37°C for 150 min. Every 30 min, the *o*-phenanthroline method was used to quantify the ferrous amounts of cultivate solution in the basolateral side (38). Ferrous lactate and FeCl_2_ (0.3 mg/mL) were used as comparison.

### 2.14 Statistical analysis

Tests were conducted triplicates, and the statistical analysis of data (mean ± standard deviation) was conducted on V.17.4 SPSS software (IBM Company, Armonk, USA). Significant difference was analyzed using Duncan’s multiple comparisons with significant consideration of *p* < 0.05.

## 3 Results and discussion

### 3.1 Separation of peptides according to ACE-inhibitory and antioxidant activity

After the hydrolysis by alcalase and trypsin assisted with ultrasound, the degree of hydrolysis of BAG-2H was 42.56% ± 3.77%. Gu et al. (29) used alcalase to hydrolyze almond protein and obtained a lower hydrolysis degree (29.22%), revealing that dual proteolysis is better at hydrolyzing bitter almond protein than alcalase single. Similar results have been obtained by Cao et al. (42). The hydroxyl radical quenching capacity, ferrous-chelating, and ACE-inhibitory abilities of BAG-2H were 43.07% ± 0.25%, 4.79 ± 0.09 mg/g, and 54.22% ± 3.00%, respectively, indicating that multifunctional peptides can be isolated from it. Alcalase and trypsin preferentially break peptide bonds that link hydrophilic and hydrophobic amino acids (2), and hydrophobic groups in proteins can be exposed after moderate ultrasound (24). The *γ*-carboxyl and ɛ-amino groups in polar amino acids of peptides, such as Asp., His, and Glu, can easily bind to ferrous ions (16), whereas hydrophobic amino acid residues have been found to remarkably restrain ACE and quench free radicals (43). Therefore, BAG-2H by alcalase and trypsin enzymolysis assisted with ultrasound showed high degree of hydrolysis and multifunction.

**Figure 1:**
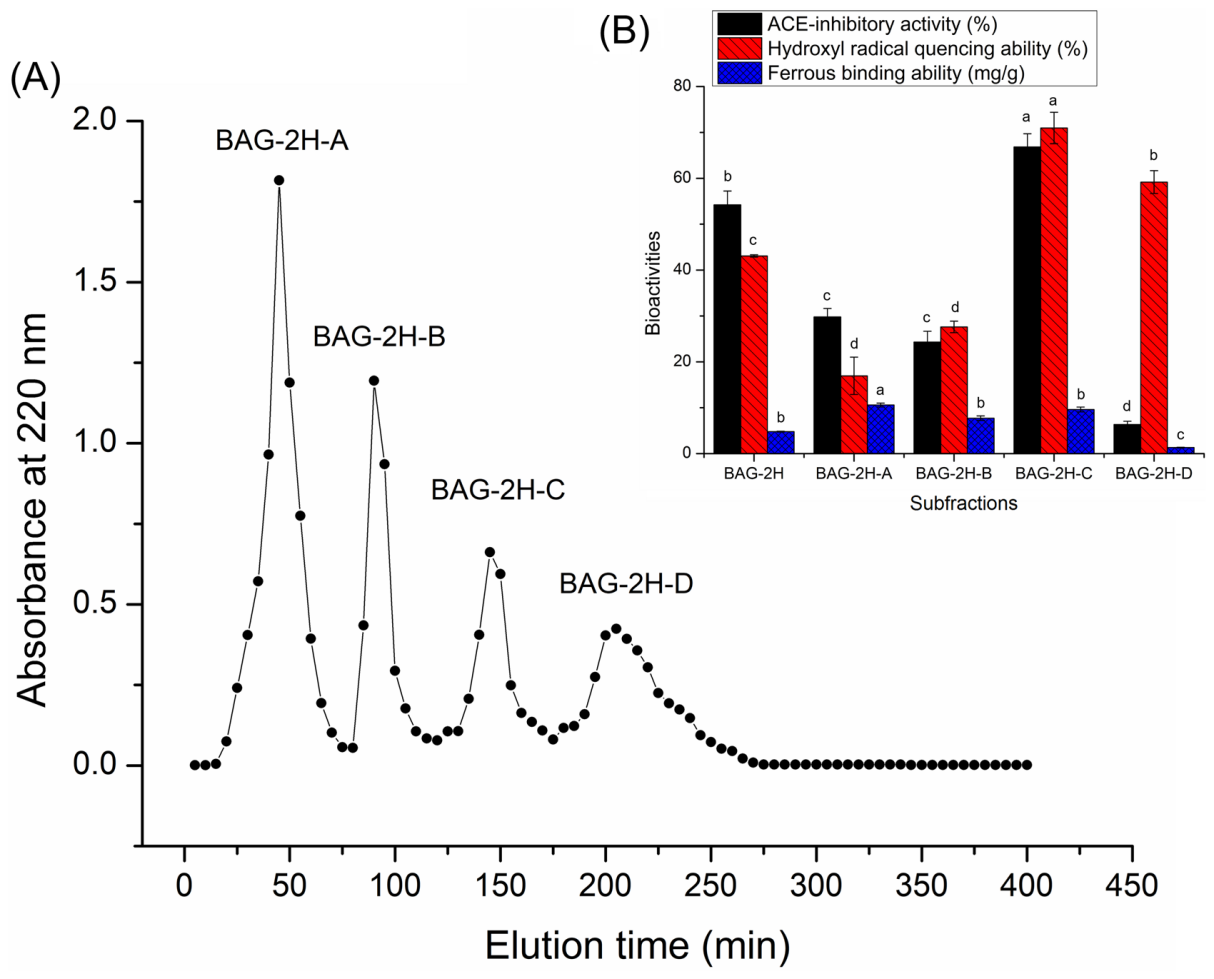
**(A)** The four subfractions (BAG-2H-A, BAG-2H-B, BAG-2H-C, and BAG-2H-D) separated from bitter almond glutelin-2 hydrolysates (BAG-2H) after Sephadex G-15 gel chromatography and **(B)** their abilities of inhibiting ACE, quenching hydroxyl radical, and chelating ferrous. Lowercase letters (a–d) above the bars represent significant difference in the same type of functionality (*p* < 0.05).

Figures 1A,B exhibit the profiles of absorbance of BAG-2H fractions (BAG-2H-A, BAG-2H-B, BAG-2H-C, and BAG-2H-D) at 220 nm against elution time during Sephadex G-15 gel chromatographic separation and the hydroxyl radical quenching, ferrous-chelating, and ACE-inhibitory abilities of those fractions, respectively. Statistical analysis showed that BAG-2H-C demonstrated better hydroxyl radical quenching capacity (70.96% ± 3.43%) and ACE-inhibitory activity (66.84% ± 2.87%) than other fractions (*p* < 0.05), although its ferrous-chelating ability (9.61 ± 0.54 mg/g, Figure 1B) was lower in comparison with that of BAG-2H-A. Hydroxyl radical quenching and ACE-inhibitory activities were the primary threshold for BAG-2H multifunctional peptide selection; therefore, BAG-2H-C’s amino acid sequence was further identified with ESI-MS/MS.

### 3.2 Identification and *in silico* screening

Peptides with >12 amino acid residues are generally not selected because of their sensitivity to digestive enzymes, low absorption rate, and potential sensitization (41). The identification results shown that there were eight polypeptides in BAG-2H-C: Asn-Gly-Gly-Gly-Asp-Met-Ser-Ala (NGGGDMSA, 707.82 Da), Pro-Val-Asp-Phe-Ala-Gly-Phe-Tyr (PVDFAGFY, 915.11 Da), Asp-Gly-Gly-Lys-Ala-Gly-Ile-Met-Thr (DGKAGIMT, 792.02 Da), Lys-Ala-Ala-Ala-Gly-Lys-Asp-Gly-Lys-Ala-Gly (KAAAGKDGKAG, 973.24 Da), Lys-Ala-Ala-Ala-Gly-Lys-Asp (KAAAGKD, 659.82 Da), Gly-Thr-Thr-Thr-Met-Ala-Pro-Ala-Ser-Ala-Lys-Gln (GTTTMAPSAKQ, 1092.38 Da), Thr-Met-Ala-Pro-Ala-Ser-Ala-Lys-Gln (TMAPSAKQ, 833.07 Da), and Gly-Ser-Gly-Gly-Glu-Glu-Ala-Ala (GSGGEEAA, 775.87 Da) (Table 1). *In silico* prediction using Peptide Ranker server and AHTpin database demonstrated that only PVDFAGFY had potential efficiency in mitigating blood pressure and oxidative stress because that its antioxidant probability and VMSS (0.51 and 0.85) were greater than the corresponding threshold values (2, 50). Supplementary Figure S1 shows the electrospray tandem mass spectra of PVDFAGFY. As NGGGDMSA, DGKAGIMT, KAAAGKDGKAG, KAAAGKD, GTTTMAPSAKQ, TMAPSAKQ, and GSGGEEAA did not show any potential antihypertension (VMSS < 0) and antioxidant activity (probability<0.4, Table 1) (2, 50), their ACE-inhibitory, antioxidant, and ferrous-binding activities were not detected. The potential sensitization and cytotoxicity of bioactive peptides can prevent their application in the food and pharmaceutical industries (17). As shown in Table 1, NGGGDMSA, PVDFAGFY, DGKAGIMT, KAAAGKDGKAG, KAAAGKD, GTTTMAPSAKQ, TMAPSAKQ, and GSGGEEAA did not show any potential allergenicity, attributed to the negative allergenic prediction results (30). Moreover, the prediction value (−0.5) by ToxinPred database confirmed that they did not have any potential toxicity (44). These results showed that NGGGDMSA, DGKAGIMT, KAAAGKDGKAG, KAAAGKD, GTTTMAPSAKQ, TMAPSAKQ, and GSGGEEAA may be used in food industry for their safety, although they lack potential antihypertensive and antioxidant activities. Safety investigation *in vivo* should be done in further study.

**Table 1:** Amino acid sequences, ACE-inhibitory capacity, ferrous-chelating activity, and *in silico* prediction on antioxidant activity, physicochemical properties, and safety of peptides identified in bitter almond glutelin-2 hydrolysates.

Peptide sequence	NGGGDMSA	PVDFAGFY	DGKAGIMT	KAAAGKD	KAAAGKDGKAG	GTTTMAPSAKQ	TMAPSAKQ	GSGGEEAA
Mass (Da)	707.82	915.11	792.02	659.82	973.24	1092.38	833.07	676.74
Matched sequence in *semen armeniacae*^a^	V.NGGGDMSA.Q	D.PVDFAGFY.T	K.DGKAGIMT.K	G.KAAAGKD.G	G.KAAAGKDGKAG.I	G.GTTTMAPSAKQ.K	T.TMAPSAKQ.G	G.GSGGEEAA.R
VMSS*^b^*	−0.86	0.51	−0.98	−0.21	−0.28	−0.70	−1.33	−0.16
Antihypertension prediction	Non-AHT	AHT	Non-AHT	Non-AHT	Non-AHT	Non-AHT	Non-AHT	Non-AHT
ACE-inhibitory activity (IC_50_: μmol/L)	ND	105.61	ND	ND	ND	ND	ND	ND
Probability ^c^	0.26	0.85	0.35	0.13	0.32	0.08	0.11	0.11
Ferrous chelating capacity (mg/g)	ND	11.76 ± 0.27	ND	ND	ND	ND	ND	ND
Hydrophobic amino acid content (%)	25.00%	62.50%	37.50%	42.86%	36.36%	36.36%	50.00%	25.00%
Hydrophobicity	−0.08	0.18	−0.06	−0.29	−0.23	−0.16	−0.19	−0.07
Amphiphilicity	0.00	0.63	0.46	1.05	1.00	0.45	0.61	0.32
Hydrophilicity	0.21	−0.79	0.25	1.07	0.91	0.00	0.10	0.66
Isoelectric point	3.80	3.80	6.19	8.94	9.72	9.11	9.11	3.80
Safety^d^								
Toxicity^e^	−0.5	−0.5	−0.5	−0.5	−0.5	−0.5	−0.5	−0.5
Allergenicity	No	No	No	No	No	No	No	No

^a^From the National Center for Biotechnology Information (NCBI); ^b^VMSS (vector machine software score) and physicochemical properties were in silico predicted using the AHTPDB database, AHT: antihypertension; ^c^the predict probability for antioxidant activity using the database Peptide Ranker server; ^d^the safety data, including potential toxicity and allergenicity, were predicted using the database ToxinPred and AlgPred, respectively. ^e^“–0.5” means non-toxic peptides. ND, not measured.

### 3.3 Structure–activity relationship analysis

**Figure 2:**
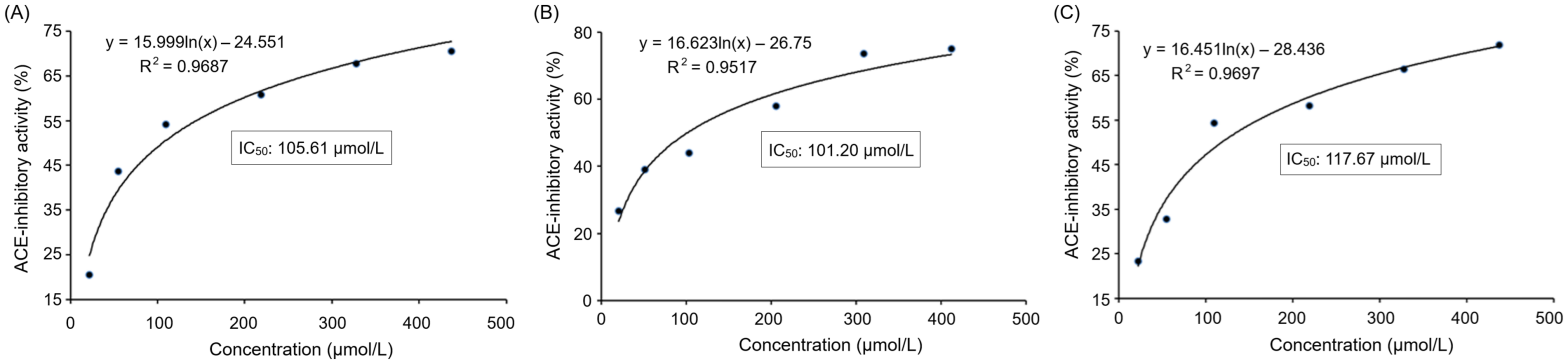
Regression analysis on ACE-inhibitory activities of PVDFAGFY **(A)**, PVDFAGFY-ferrous chelate **(B)**, and PVDFAGFY treated by gastrointestinal digestion **(C)**. IC_50_ means the amount of the samples required to inhibit half activity of ACE.

The inhibitory effect of PVDFAGFY on ACE was a logarithmic function with its concentration (Figure 2A), from which the IC_50_ value (105.61 μmol/L) was calculated. As decrease in IC_50_ value indicates increase in ACE inhibition capacity of peptides (50), PVDFAGFY showed better ACE-inhibitory ability than peptides GCHHY from millet bran glutelin-2 (IC_50_:147 μmol/L) (34) and VIPTEPPHA from Faba beans (IC_50_:259.7 μmol/L) (45) but exhibited lower ability than Captopril (IC50:0.14 μmol/L) which is widely used for antihypertension (9). Furthermore, the results in Table 2 show that chemically synthesized PVDFAGFY had significant proton-providing capacity (0.401), and clearance rate of hydroxyl (96.58%), ABTS (72.78%), and superoxide radicals (72.31%).

**Table 2:** Antioxidant activity of bitter almond glutelin-2 hydrolysates fraction C (BAG-2H-C) and antihypertensive peptides identified in BAG-2H-C at 100 μg/ mL with glutathione as comparison.

Samples	ABTS^+^ scavenging activity (%)		·OH scavenging activity (%)		Superoxide radical scavenging ability (%)		Ferric reducing ability	
	Before gastrointestinal	After gastrointestinal	Before gastrointestinal	After gastrointestinal	Before gastrointestinal	After gastrointestinal	Before gastrointestinal	After gastrointestinal
BAG-2H-C	60.05% ± 2.15%b	ND	43.07% ± 0.25%c	ND	41.41% ± 4.29%c	ND	0.293 ± 0.012c	ND
PVDFAGFY	72.78% ± 4.67%a	75.37% ± 3.18%a	96.58% ± 3.77%a	92.00% ± 4.82%a	72.31% ± 0.47%b	69.08% ± 2.75%a	0.401 ± 0.009b	0.385 ± 0.015b
PVDFAGFY-Fe	35.18% ± 1.16%c	ND	79.37% ± 4.21%b	ND	69.55% ± 3.41%b	ND	0.225 ± 0.012d	ND
Glutathione	79.25 ± 1.67%a	ND	95.56% ± 1.38%a	ND	90.52% ± 4.97%a	ND	0.675 ± 0.019a	ND

Different lowercase letters (a–d) in the same column mean significant difference (*p* < 0.05); ND, not measured.

Prior studies have confirmed that a peptide will be better at inhibiting ACE if special residues, including Phe, Tyr, Arg, Gln, Val, and Pro, are in its *C*-terminal tripeptide or *N*-terminal (12, 22, 36). Now, the strong affinity between ACE and some special chemical groups in ACE-inhibitory peptides, including guanidyl, sulfhydryl, *γ*-hydroxyl, and *ε*-amino groups, has been revealed by molecular docking and other virtual technologies (22, 46, 47). Therefore, the restraining ability of PVDFAGFY toward ACE was predominately ascribed to the Pro, Tyr, Phe, and Val residues.

Alternatively, the cyclic imino group of Pro and phenolic hydroxyl group in Tyr or Phe can quickly quench free radicals, showing excellent antioxidant ability (43). The γ-carboxyl group in Asp can inhibit free radicals’ chain reaction via chelating metal ions which are catalyst for oxidation reaction (2). Thus, the remarkable antioxidant activity of PVDFAGFY was primarily due to the Tyr, Pro, Phe, Val, and Asp residues. Broad bean antioxidant peptide VSRRFIYYL and apricot antioxidant peptides YLSF and LPSYVN were rich in Tyr, Pro, or Phe and offered excellent antioxidant activity (1, 2).

As shown in Table 1, PVDFAGFY offered a remarkable ability to chelate ferrous ions (11.76 ± 0.27 mg/g), which was higher than that of millet bran globulin peptide SELE (7.93 mg/g) (31) and scallop skirts peptide FEDPEFE (9.39 mg/g) (21). The Asp., Val, Tyr, Phe, and Gly in PVDFAGFY can effectively bind to ferrous ions (16, 20). The *γ*-carboxyl group in Asp and phenolic hydroxyl groups in Tyr and Phe can form ionic bond with ferrous ions (17). Ascribed to the excellent metal ion-chelating capacity, Gly is an ideal ingredient for iron or calcium supplement (45). In addition, the carbonyl group and amido bond in PVDFAGFY have been proven to show ferrous ion-binding ability (38).

Physicochemical characters of peptides could be specific, which are dependent on their amino acid sequence (41). Prior studies have shown that peptides with high hydrophobicity can effectively prevent oxidant and hypertension by binding to Keap1 and ACE, respectively (5, 9). A high hydrophilicity indicates that peptides have relatively strong affinity for metal ions (19). Furthermore, the amphiphilicity represents both hydrophilicity and hydrophobicity (2). As shown in Table 1, the hydrophobicity, amphiphilicity, and hydrophilicity of PVDFAGFY were 0.18, 0.63, and − 0.79, respectively, corresponding to its high antioxidant, ACE-inhibitory, and ferrous-binding activities (Tables 1, 2 and Figure 2A). In addition, ferrous fortification of PVDFAGFY may be reduced at its isoelectric points of 3.80 because of their lowest surface charge (18).

### 3.4 Inhibition mechanisms toward ACE and Keap1

#### 3.4.1 Molecular docking of Keap1 with PVDFAGFY

**Figure 3:**
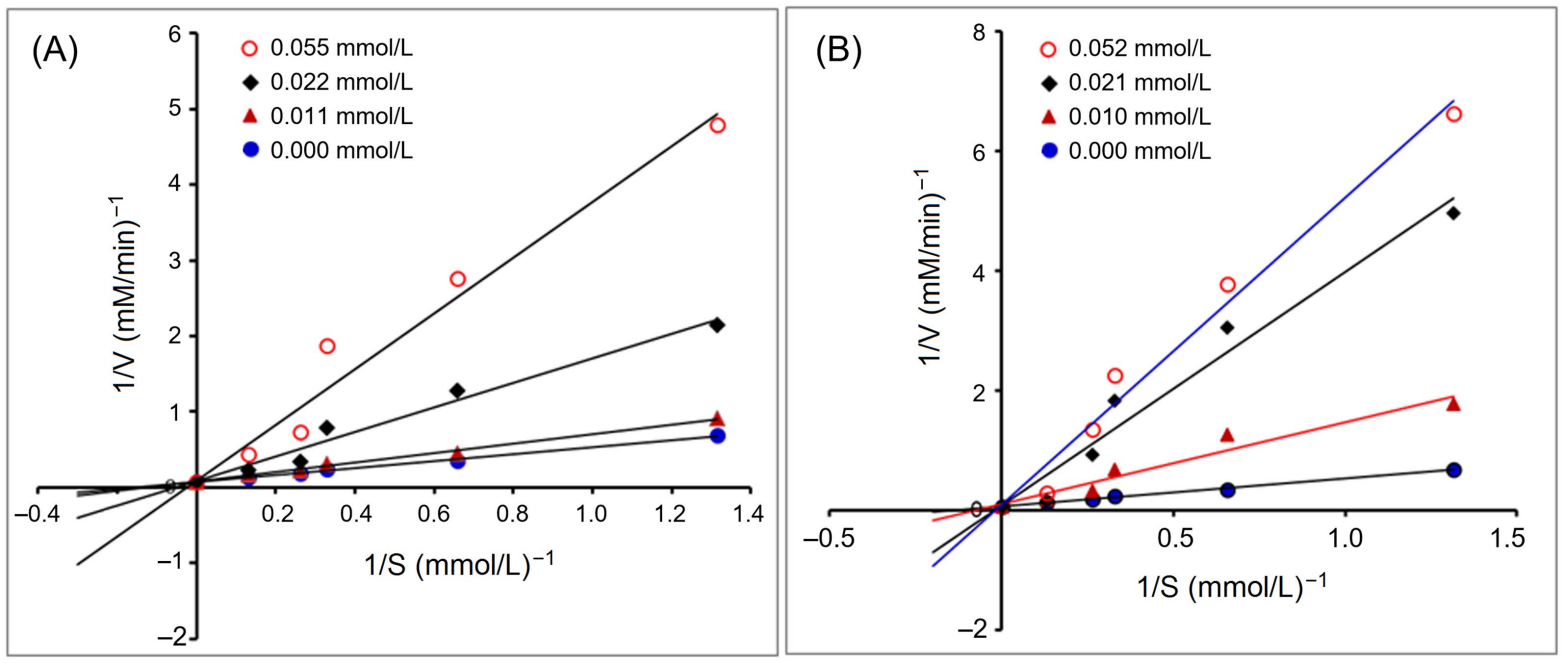
Interaction models for hydrogen bonding interactions within binding details of PVDFAGFY with Keap1 (PDB ID: 2FLU) **(A)** and ACE (PDB: 1O8A) **(B)**. The yellow dotted line means hydrogen bond.

Antioxidants can lower oxidative pressure via inhibiting the interactions between Nrf2 and Keap1 and improving the expression of cytoprotective genes and antioxidant enzymes (35). As shown in Figure 3A and Table 3, PVDFAGFY can bind to six residues of Keap1 (Val608, Gly367, Val512, Val606, Ile559, and Ala510) via 11 short hydrogen bonds (2.02–3.14 Å). The six residues, especially Val512 and Val606, have been proven to be main resides through which Keap1 binds to Nrf2, and binding to them can apparently lower the inhibiting ability of Keap1 on the transcription of Nrf2 and thus mitigate cellular oxidative damage (6, 48). Moreover, the T-score for the domain docking model of Keap1 with PVDFAGFY (11. 00, Table 3) was much higher than the threshold (6.0) (22), suggesting that PVDFAGFY has relatively strong affinity with the Kelch domain of Keap1 and consequently can inhibit the interaction between Nrf2 and Keap1 (2). However, specific mechanisms require more studies.

**Table 3:** Interactions between active sites of ACE or Keap1 with PVDFAGFY identified in bitter almond glutelin-2 hydrolysates using molecular docking simulation.

Targets	T-score	C-score	Residues and the length of hydrogen bonds	Hydrophobic interaction
ACE (PDB-108A)	10.65	5.00	Tyr523: 2.91 Å; Tyr520: 1.97 Å; Glu376: 2.88 Å; Gln281: 2.42 Å, 2.76 Å; Ala354: 1.99 Å; His353: 2.89 Å; Lys511: 3.12 Å	Ser355, Gln281, Glu376, Lys454, Asp453, Thr282, Val379, Val380, His383, His353, Ala354, Val518, Asn277, Phe391
Keap1 (ID:2FLU)	11.00	4.00	Val608: 3.14 Å; Gly367: 2.85 Å; Val606: 2.02 Å; Val512: 3.02 Å; Val512: 2.98 Å; Ile559: 2.76 Å	Glu79, Val420, Gly367, Val418, Val514, Val606, Val418

#### 3.4.2 Molecular docking of ACE with PVDFAGFY

Peptides that can affect the substrate-linking center (S1, S2, and S1’ pockets) or catalytic triad (containing a zinc tetrahedron) of ACE are better at inhibiting ACE (36). The molecular docking results in Figure 3B and Table 3 revealed that six key residues in substrate-linking center of ACE, including Tyr523, Tyr520, Gln281, Lys511, Ala354, and His353, can be bound by PVDFAGFY via short hydrogen bonds (1.97–3.12 Å). Among them, Lys511, Gln281, His353, and Tyr520 are key residues of S2 pocket in ACE’s substrate-linking center (49). The Ala354 and Tyr523 are crucial residues of the S1 pocket (50). Binding to one of these key residues in ACE indicates that peptides can competitively link to the substrate-linking center and show excellent ACE-inhibitory capacity (9). Moreover, PVDFAGFY can bind to 14 residues of ACE by hydrophobic interactions (Figure 3B and Table 3). The His383 is component of the zinc tetrahedron (11). Therefore, PVDFAGFY can restrain ACE by affecting the substrate-linking sites of ACE, or impacting its zinc tetrahedron. In addition, the distance of hydrogen bonds between PVDFAGFY and ACE was short (1.97–3.12 Å), and the T-scores and C-scores for the domain docking model of ACE with PVDFAGFY (10.65 and 5.00, Table 3) were much higher than the threshold (6.0 and 4.0, respectively) (22), corresponding to its high ACE-inhibitory activity.

#### 3.4.3 ACE-inhibitory kinetics

**Figure 4:**
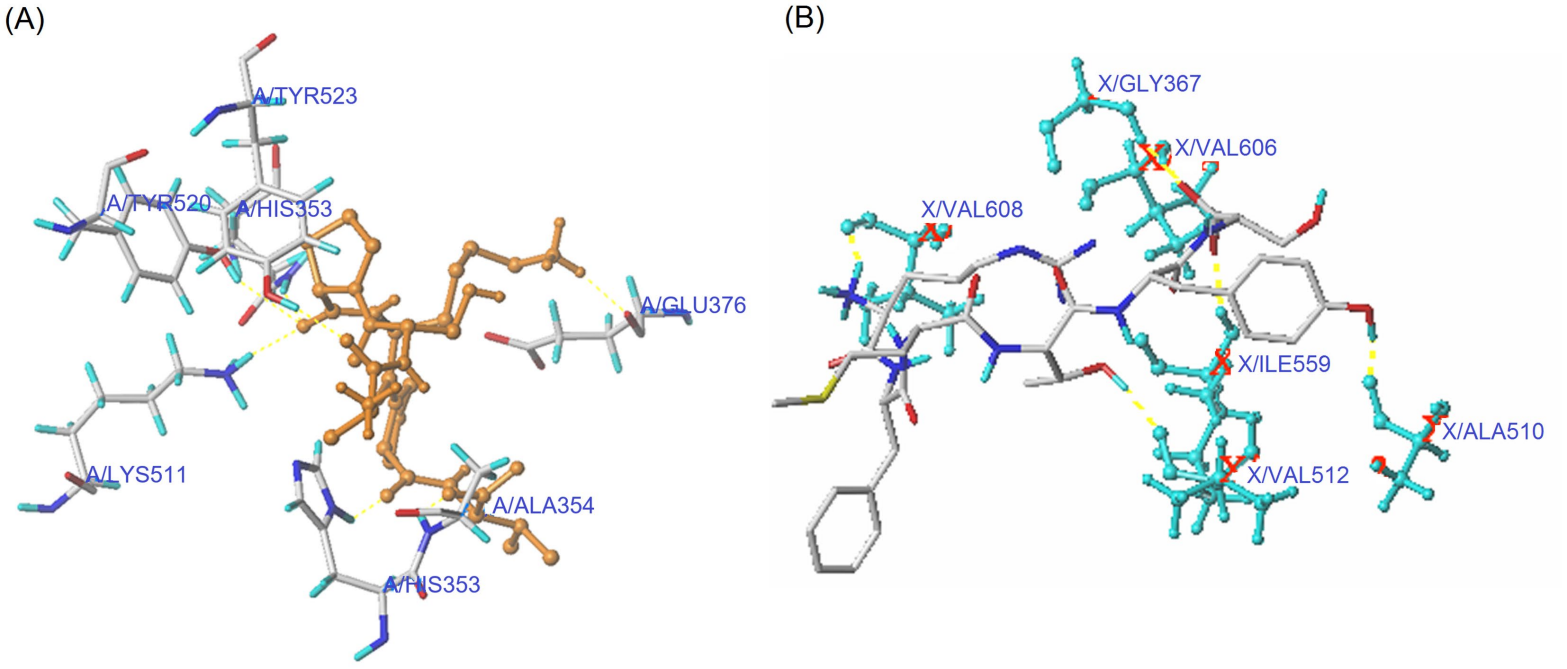
Lineweaver–Burk plots of the ACE inhibition for PVDFAGFY **(A)** and PVDFAGFY-ferrous chelate **(B)**.

Figure 4A depicts the effect of addition of PVDFAGFY on hippuric acid’s production rate from the hydrolysis of HHL by ACE. The Michaelis–Menten kinetic curves reveal that the maximum velocity (*V*_max_) was not altered but the *K_m_* value was increased as the dose of PVDFAGFY increased, verifying that addition of PVDFAGFY lowered the production rate of hippuric acids via competitively binding to the binding center of substrate in ACE and restraining its affinity to the substrate (HHL). Correspondingly, Figure 3B showed that PVDFAGFY formed short hydrogen bonds with active residues in ACE’s active center S1 and S2. As peptides with a competitive inhibit model are better at inhibiting ACE (50), PVDFAGFY shows excellent ACE inhibition capacity (IC_50_:105.61 μmol/L, Table 1).

### 3.5 Chelation patterns between PVDFAGFY and ferrous ions

**Figure 5:**
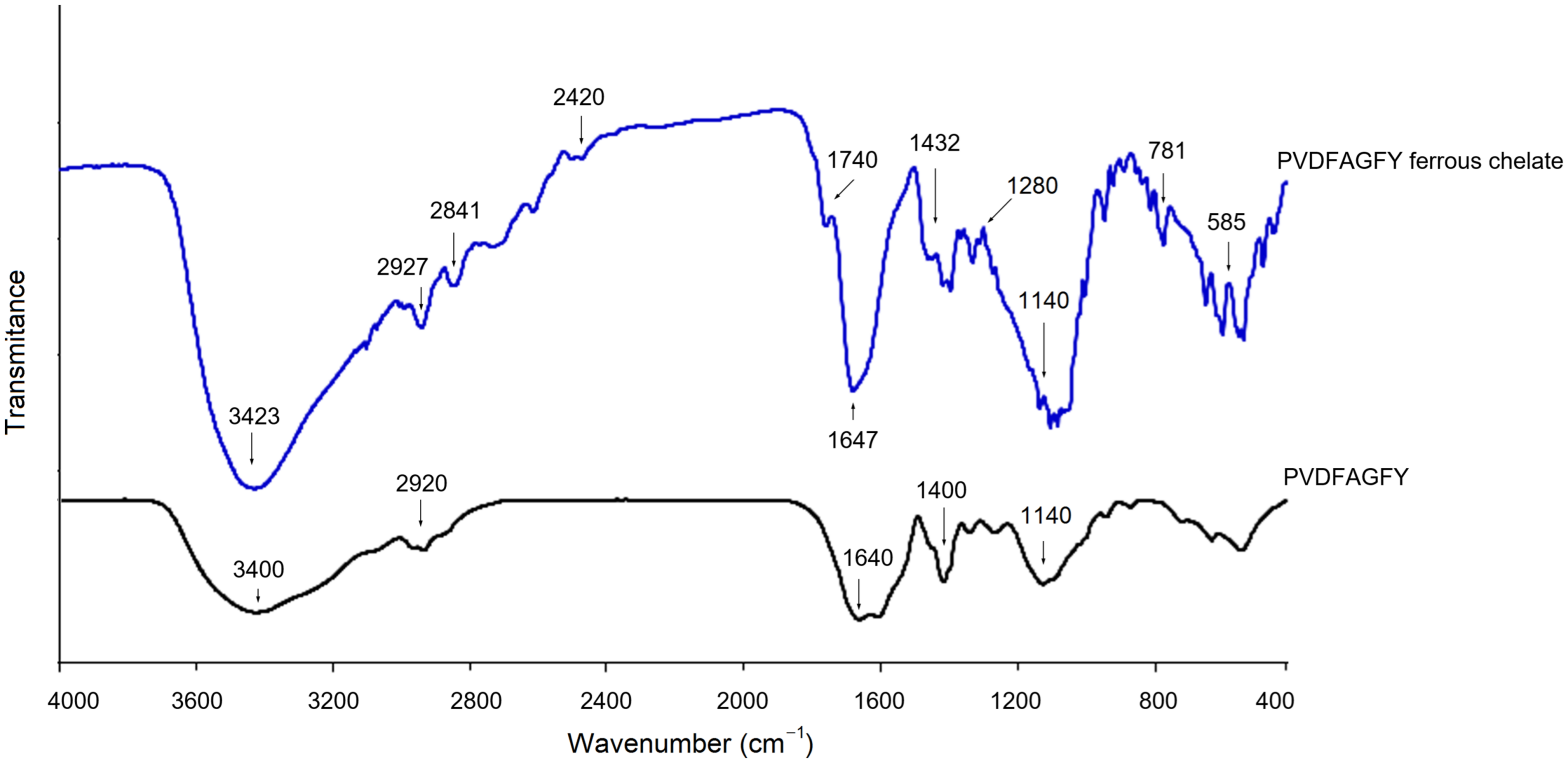
Fourier-transformed infrared spectra of PVDFAGFY and PVDFAGFY-ferrous chelate.

The ferrous-chelating patterns of PVDFAGFY are shown in Figure 5. Slight difference can be seen between the FI-IR spectra of PVDFAGFY-ferrous chelate and PVDFAGFY. After ferrous chelation, the branded peak at 3400 cm^−1^ in the spectra of PVDFAGFY shifted to 3,423 cm^−1^, indicating the interactions between ferrous ions and the hydroxyl groups in PVDFAGFY (45). The blueshift (from 1,640 to 1,647 cm^−1^) appeared in the spectrum of PVDFAGFY-ferrous chelate confirmed the binding force of the carbonyl groups of the amide band I to ferrous ions (21). Moreover, the carbon–nitrogen bond in amide band III of PVDFAGFY cheated ferrous ions because there was a new peak appeared at 1740 cm^−1^ in the spectrum of PVDFAGFY-ferrous chelate (41). The phenolic hydroxyl group of Tyr and Phe in PVDFAGFY bound to ferrous ions because there were new peaks at 1280 and 2,841 cm^−1^ (representative of the aromatic acids and methyl groups in the benzene ring) (18). In addition, the new peak appeared at 1740 cm^−1^ after chelation of PVDFAGFY confirmed the linkage of ferrous ions with *γ*-carboxyl group in Asp (17). Thus, ferrous ions were chelated by the carboxyl, phenolic hydroxyl, and amido groups of PVDFAGFY, which was consistent with the results of Ding et al. (16) and Hu et al. (19).

### 3.6 Influences of ferrous chelation on bioactivities of PVDFAGFY

As shown in Figures 2A, 3B, the ACE-inhibitory IC_50_ value of PVDFAGFY-ferrous chelate (101.20 μmol/L) was similar to that of PVDFAGFY (*p* > 0.05), highlighting the ACE inhibition capacity of PVDFAGFY was not notably altered by ferrous chelation. Moreover, as shown in Figure 4B, addition of PVDFAGFY-ferrous chelate increased the *Km* value of the Michaelis–Menten kinetic curves but did not change the *V*_max_, verifying PVDFAGFY-ferrous chelate is a competitive ACE inhibitor (51). Therefore, the ACE-inhibitory activity and model of PVDFAGFY were not changed by ferrous chelation. As PVDFAGFY can form short hydrogen bonds with six active sites (Tyr523, Tyr520, Gln281, Lys511, Ala354, and His353) in ACE’s substrate-linking center (Table 3 and Figure 3B), it is difficult for ferrous chelation to completely restrain the interactions between these key residues and PVDFAGFY (47). Similar trend was obtained by Xu et al. (31).

As shown in Table 2, ferrous chelation remarkably decreased the antioxidant activity of PVDFAGFY, because the ferric reducing ability, and hydroxyl radical and ABTS cation quenching activity of PVDFAGFY-ferrous chelate were lower than those of PVDFAGFY (*p* < 0.05). Ferrous chelation changed the ability of PVDFAGFY to absorb electrons or give protons and thus lowered its oxidation resistance (3), even though PVDFAGFY-ferrous chelate exhibited considerable antioxidant activity, including ferric reducing ability (0.225), quenching abilities on hydroxyl (79.37%) and superoxide radicals (69.55%, Table 2), and excellent ferrous ions (the catalyst of oxidation reaction) chelation ability (11.76 mg/g, Table 1), suggesting its potential ability to restrain oxidation (45).

### 3.7 Gastrointestinal stability

As shown in Figure 2C, the PVDFAGFY treated by gastrointestinal digestion showed an equivalent ACE inhibition IC_50_ value (117.67 μmol/L) with that of PVDFAGFY (*p* > 0.05). Moreover, the antioxidant activity of PVDFAGFY treated by gastrointestinal digestion was not significantly lower than those of untreated PVDFAGFY (*p* > 0.05), including ferric reducing ability and quenching abilities on ABTS cation, hydroxyl, and superoxide radicals (Table 2). These findings showed that the antioxidant activity and ACE-inhibitory capacity of PVDFAGFY were stable during gastrointestinal digestion. Pro residue has been proven to notably increase peptides’ gastrointestinal stability because of its rigid imino ring (34). Moreover, branched amino acid (Val, Ile, and Leu) can increase the steric hindrance of peptides, which is conducive to the peptides’ stability (12). Thus, the Pro and Val residues in PVDFAGFY were mainly responsible for its gastrointestinal stability.

**Figure 6:**
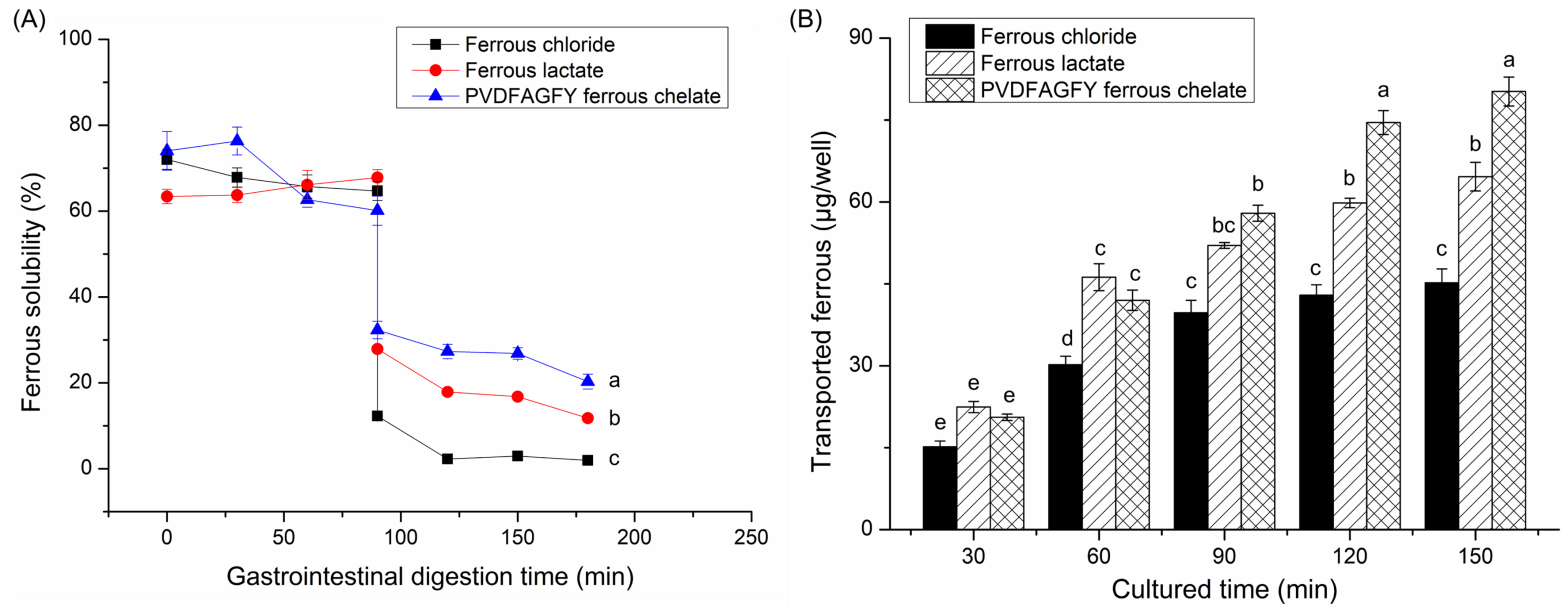
**(A)** Ferrous solubility of the ferrous chloride, ferrous sulfate, and PVDFAGFY-ferrous chelate against simulated gastrointestinal digestion and **(B)** the transport ferrous amount across Caco-2 cell monolayers by PVDFAGFY-ferrous chelate, ferrous lactate, and FeCl_2_. Different lowercase letters (a–e) on the bars or near the lines are representative of significant differences (*p* < 0.05).

As shown in Figure 6A, during gastric digestion (0–90 min), the ferrous solubility of ferrous lactate, PVDFAGFY-ferrous chelate, and ferrous chloride was relatively stable because ferrous ions do not oxidize in acidic environments (19); however, their ferrous solubility apparently decreased at 91–180 min (*p* < 0.05) because soluble ferrous ions were converted to insoluble compounds when the pH value increased sharply (45). Similar results were found in prior reports (30, 41). More importantly, from 91 to 180 min, the ferrous solubility of PVDFAGFY-ferrous chelate was much higher than those of ferrous lactate and ferrous chloride (*p* < 0.05), indicating that PVDFAGFY is better at improving ferrous gastrointestinal stability, mainly ascribed to the excellent ferrous-binding ability (11.76 mg/g, Table 1) and gastrointestinal stability of PVDFAGFY (Figure 2C) (20). However, more studies were needed to investigate the special effect of gastrointestinal hydrolysis on the interactions between ferrous ions and PVDFAGFY.

### 3.8 Transmembrane absorption of ferrous ions

The ability of PVDFAGFY-ferrous chelate to improve the ferrous transmembrane absorption was higher than that of ferrous chloride (*p* < 0.05, Figure 6B). During 120–150 min, PVDFAGFY-ferrous chelate was better at transporting iron across the monolayer of Caco-2 cells than ferrous lactate (which is widely used as an adjunct to treatment anemia) (*p* < 0.05), indicating its potential application as iron supplements (19). One reason for this is the excellent ferrous-chelating ability (Table 1) and gastrointestinal stability of PVDFAGFY-ferrous chelate (Figure 6A). Furthermore, ferrous ions are absorbed mainly through the ion channel pathway (containing multiple carriers, enzymes), while binding to PVDFAGFY may alter the *in vivo* absorption way of ferrous ions (16). Prior studies have found that transporter (PepT1), interstitial cells, and endocytosis channels are the main absorption way of peptide-ferrous chelate, which are faster and energy consumption (20, 50). The special absorption way of PVDFAGFY-ferrous chelate needs further study.

## 4 Conclusion

A safe multifunctional octapeptide: PVDFAGFY, was isolated, identified, and screened from *semen armeniacae* glutelin-2 hydrolysates by combining *in vitro* and *in silico* ways. The capacities of PVDFAGFY to restrain ACE, chelate ferrous ions, and quench hydroxyl radical were IC_50_:105.61 μmol/L, 11.67 mg/g, and 97.67%, respectively. PVDFAGFY restrained ACE via competitively linking to its catalytic (His383) and/or crucial binding sites (Gln281, Lys511, Tyr523, Tyr520, or Ala354), and it can inhibit the Keap1-Nrf2 interaction by binding to 6 residues of Keap1. Ferrous ions were predominantly chelated by the carboxyl, phenolic hydroxyl, and amide groups in PVDFAGFY. Moreover, PVDFAGFY-ferrous chelate showed the same ACE-inhibitory model and activity to that of PVDFAGFY (*p* > 0.05) but demonstrated lower hydroxyl and ABTS radical quenching capacity and ferric reducing ability (*p* < 0.05). The gastrointestinal stability and transmembrane absorption across the Caco-2 cell monolayer of ferrous ions were increased by PVDFAGFY. These findings show that PVDFAGFY may be exploited as ingredients of hypotensive, antioxidant, and/or iron supplementary agents, but its specific effect on Keap1-Nrf2-ARE system and *in vivo* antioxidant and hypotensive efficiencies need further study.

## Data Availability

The original contributions presented in the study are included in the article/Supplementary material, further inquiries can be directed to the corresponding authors.

## Footnotes

^1^ncbi.nlm.nih.gov/guide/

^2^ https://webs.iiitd.edu.in/raghava/ahtpin/penta_design.php

^3^ http://distilldeep.ucd.ie/PeptideRanker/

^4^ imtech.res.in/raghava/algpred/

^5^ imtech.res.in/raghava/toxinpred/
