# Supplementary material for: Purification, identification, and in silico screening of a multifunctional octapeptide from semen armeniacae glutelin-2 hydrolysates: restraining mechanisms to Keap1 and ACE, stability, and ferrous-transport efficiency
Source: Front Nutr. 2025 Apr 4;12:1571161. doi: 10.3389/fnut.2025.1571161 (PMC12005989; doi:10.3389/fnut.2025.1571161)
Supplement: Supplementary file 1 [file Image_1.pdf]

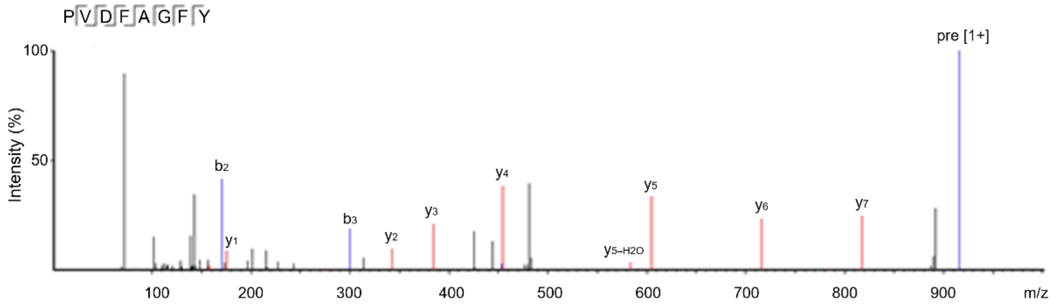

Figure S1. The secondly electrospray tandem mass spectra of peptide PVDFAGFY identified in bitter almond glutelin-2 hydrolysates.
